# Trichostatin A Enhances the Apoptotic Potential of Palladium Nanoparticles in Human Cervical Cancer Cells

**DOI:** 10.3390/ijms17081354

**Published:** 2016-08-19

**Authors:** Xi-Feng Zhang, Qi Yan, Wei Shen, Sangiliyandi Gurunathan

**Affiliations:** 1 College of Biological and Pharmaceutical Engineering, Wuhan Polytechnic University, Wuhan 430023, China; zhangxf9465@163.com (X.-F.Z.); yanyy75@163.com (Q.Y.); 2 Key Laboratory of Animal Reproduction and Germplasm Enhancement in Universities of Shandong, College of Animal Science and Technology, Qingdao Agricultural University, Qingdao 266109, China; shenwei427@126.com; 3 Department of Stem Cell and Regenerative Biology, Konkuk University, Seoul 143-701, Korea

**Keywords:** cervical cancer, palladium nanoparticles, trichostatin A, cell viability, oxidative stress, mitochondrial membrane potential, caspases, apoptosis

## Abstract

Cervical cancer ranks seventh overall among all types of cancer in women. Although several treatments, including radiation, surgery and chemotherapy, are available to eradicate or reduce the size of cancer, many cancers eventually relapse. Thus, it is essential to identify possible alternative therapeutic approaches for cancer. We sought to identify alternative and effective therapeutic approaches, by first synthesizing palladium nanoparticles (PdNPs), using a novel biomolecule called saponin. The synthesized PdNPs were characterized by several analytical techniques. They were significantly spherical in shape, with an average size of 5 nm. Recently, PdNPs gained much interest in various therapies of cancer cells. Similarly, histone deacetylase inhibitors are known to play a vital role in anti-proliferative activity, gene expression, cell cycle arrest, differentiation and apoptosis in various cancer cells. Therefore, we selected trichostatin A (TSA) and PdNPs and studied their combined effect on apoptosis in cervical cancer cells. Cells treated with either TSA or PdNPs showed a dose-dependent effect on cell viability. The combinatorial effect, tested with 50 nM TSA and 50 nMPdNPs, had a more dramatic inhibitory effect on cell viability, than either TSA or PdNPs alone. The combination of TSA and PdNPs had a more pronounced effect on cytotoxicity, oxidative stress, mitochondrial membrane potential (MMP), caspase-3/9 activity and expression of pro- and anti-apoptotic genes. Our data show a strong synergistic interaction between TSA and PdNPs in cervical cancer cells. The combinatorial treatment increased the therapeutic potential and demonstrated relevant targeted therapy for cervical cancer. Furthermore, we provide the first evidence for the combinatory effect and cytotoxicity mechanism of TSA and PdNPs in cervical cancer cells.

## 1. Introduction

Cervical cancer is a common cancer and ranks seventh overall, among all types of cancer in women. Data, recorded in 2012, showed that 528,000 new cases of cervical cancer were identified, and 266,000 resulted in death worldwide; this accounted for 7.5% of all female cancer-related deaths [1]. The American Cancer Society reported that 12,990 new cases of invasive cervical cancer will be diagnosed in 2016, and 4120 women will die from the disease [2]. The current position of cancer treatment highlights the importance of developing new effective anticancer therapeutic molecules and protocols for preventing cervical cancer [2]. The occurrence and progression, of cervical cancer are believed to be related to abnormal genetic and epigenetic regulation, including phosphorylation and acetylation of histone H3, global DNA hypo-methylation and hyper-methylation of tumor suppressor genes [3,4]. The acetylation and de-acetylation of the *N*-terminal histone tails, by specific histone acetylases and deacetylases, are involved in gene regulation [5].

Histone acetyl transferase (HAT) and histone deacetylase (HDAC) are two classes of enzymes involved in maintaining chromatin structure and function through acetylation and deacetylation, respectively [6]. An imbalance, between these two enzymes, causes several dysfunctions, including an anti-proliferative effect, mitotic defects, cell growth, gene silencing, malignant transformation and aberrant cell signaling [6,7,8,9]. HDAC inhibitors are a new class of potent HDAC-specific inhibitors that lead to tumor cell arrestin a variety of cancers [10,11]. Mishra et al. (2001) reported that HDAC inhibitors are able to reduce cell survival in human breast cancer cells through remodeling of the human epidermal growth factor receptor 2 (HER2) promoter and HER2 expression [12]. Trichostatin A (TSA) is one of several inhibitors, which shows a potential therapeutic effect in various types of cancer cells, when combined with radiotherapy or chemotherapy [13,14]. TSA causes apoptosis through the inhibition of cell viability, as well as proliferation and activation of apoptosis-related proteins in a variety of cancer cells, including human gastric, ovarian and small cell lung cancer cells [15,16,17]. TSA shows potential as an inducer of apoptosis. However, few reports describe the effect of a combination of TSA and nanoparticles on apoptosis. Several studies reported that nanoparticles play a crucial role in cancer therapeutics [18,19].

The synthesis of nano-sized noble metals possessing unique physical, chemical and biological properties is gaining considerable interest [20,21]. Palladium nanoparticles (PdNPs), among several other metal nanoparticles, play a major role in the industry. This is due to their heterogeneous and homogeneous catalytic properties, high surface-to-volume ratio and high surface energy and nano architectonics [22,23,24]. A unique property of Pd complexes of polyamides containing sulfones is their high antimicrobial potency [25]. PdNPs exhibited a significant synergistic, high efficacy effect, compared to the sum of the individual efficacies of chemotherapy and photothermal therapy [26]. PdNPs showed high cytotoxicity against five different human cancer cell lines [27]. Additionally, PdNPs induce concentration-dependent cytotoxicity, apoptosis and alterations in the release and expression of numerous cytokines [28,29] and induction of apoptosis and autophagy in human ovarian cancer cells [30]. PdNPs have been used as antibacterial, antifungal and anticancer agents, but their effect in cancer studies remains elusive. Therefore, analyzing the effective role of PdNPs in biological and biomedical applications is essential. Significant numbers of studies have reported the synthesis and characterization of PdNPs by using chemical methods; however, the significance of biological molecule-mediated synthesis of PdNPs and cytotoxicity-mediated mechanisms by PdNPs is not well known. Therefore, the synthesis of biocompatible, non-toxic, environmentally-friendly PdNPs is essential. The resulting biocompatible PdNPs will be useful and effective for cancer studies with chemotherapeutic agents.

Chemotherapy has advanced in cancer therapy using a variety of targeted drugs. The antitumor efficacies of current therapies are limited due to the high degree of cancer clonal heterogeneity and drug resistance [31]. A single therapeutic agent is not able to eradicate the cancer; the use of combinatorial therapy, which could inhibit multiple targets or redundant pathways simultaneously, is essential and inevitable [31]. For example, a combination of vascular endothelial growth factor (VEGFR) antibody DC101 and vinblastine results in full and sustained regression of large, established tumors in neuroblastoma xenograft models [32]. Combinations of TSA and quercetin (10–40 µM) induce cell death in human leukemia HL-60 cells [33]. The combination of Akt inhibitor and TCA potentiates apoptosis in ovarian carcinoma cell lines by increasing the activation of the caspase-8-dependent pathway and the mitochondria-mediated cell death pathway [34]. Targeting signaling pathways, such as the RAS/RAF/MEK/ERKandPI3K/Akt/Src/mTOR pathways, is important for combination therapy. These are the main pathways for extracellular-mediated cell survival, cell proliferation, differentiation and development [31,34,35,36,37,38,39,40].

Currently, several approaches have been developed to improve the activity and overcome multi-drug resistance; new combinations of novel anticancer drugs, which specifically target cancer cells, reduce the side effects of chemotherapy [2]. Combinations of chemotherapeutic drugs are most effective because each drug is effective against a different mechanism, which decreases drug-resistant cancer cells. A considerable number of studies was performed with a combination of various chemotherapeutic agents in different kinds of cancer cells. None of the studies has pursued the combination of TSA and palladium nanoparticles. Particularly, the combination of chemotherapeutic agents with nanoparticles for the treatment of cervical cancer has not been reported. This study was based on three main objectives. The first aim was to synthesize and characterize PdNPs by using a biomolecule called saponin. The second aim was to investigate the growth-inhibitory effects of HDAC inhibitor, TSA and palladium nanoparticles in cervical cancer cells. The final objective was to evaluate the mechanistic effect of a TCA and palladium nanoparticle combination, on apoptosis.

## 2. Results and Discussion

### 2.1. Synthesis and Characterization of Palladium Nanoparticles (PdNPs)

**Figure 1:**
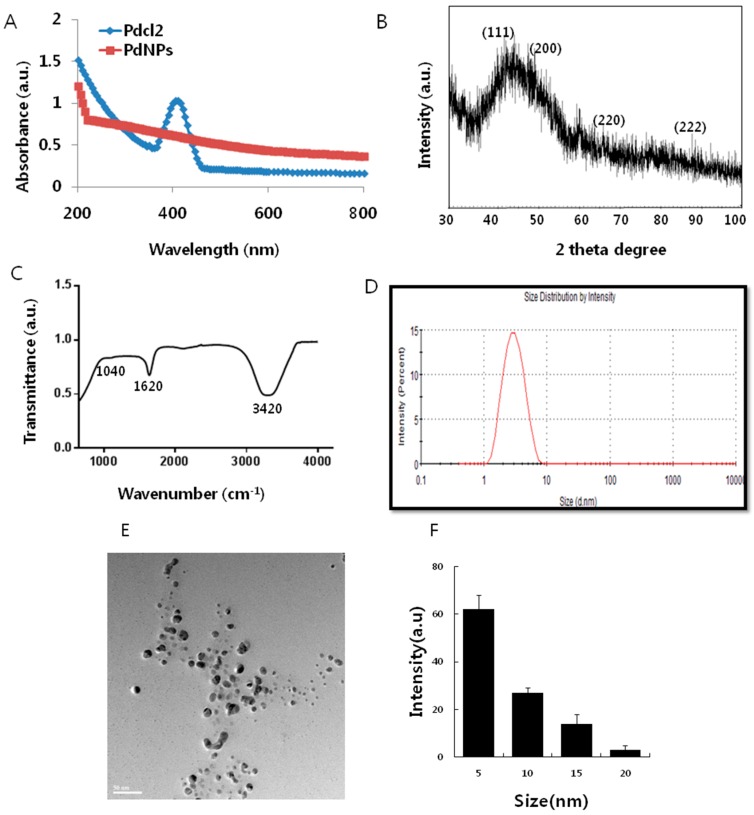
Synthesis and characterization of palladium nanoparticles (PdNPs). (**A**) Ultraviolet-visible spectroscopy (UV-VIS) spectra of PdNPs. Pdcl2: Palladium(II) chloride; (**B**) X-ray diffraction (XRD) pattern of PdNPs; (**C**) Fourier transform infrared spectroscopy (FTIR) spectra of PdNPs; (**D**) size distribution analysis of PdNPs by dynamic light scattering (DLS); (**E**) Transmission electron microscopy (TEM) images of PdNPs; (**F**) size distributions based on TEM images of PdNPs, ranging from 5–20 nm.

The synthesis of the PdNPs was performed at 60 °C, using an aqueous solution of PdCl_2_ and saponin, which served as a reducing and stabilizing agent. Figure 1A shows the ultraviolet-visible spectroscopy (UV-VIS)spectra of the aqueous PdCl_2_ solution and the Pd colloidal suspensions, after reduction. The UV-VIS spectrum of the PdCl_2_ reference sample showed a peak at 415 nm due to the absorption of Pd(II) ions. The peak at 415 nm was absent in the reduced samples. A broad continuous absorption was observed, which indicated complete reduction of Pd(II) ions to PdNPs [30]. Further characterization was carried out by X-ray diffraction (XRD). The results showed four peaks at 40.0°, 50.0°, 65.0° and 88.0°, which corresponded to reflections from the (1 1 1), (2 0 0), (2 2 0), (3 1 1) and (2 2 2) planes of the face-centered cubic (fcc) lattice, respectively [30,41,42]. The most intensive and predominant peak of the PdNPs crystals was observed at 50.0°, which corresponded to (1 1 1) planes. The broad peak at 40.0° is the characteristic peak of the (1 1 1) indices of Pd(0), which is a face-centered cubic structure (Figure 1B).

Fourier transform infrared spectroscopy (FTIR) analysis confirmed the involvement of the biological molecule in the reduction processes. As shown in Figure 1C, the major absorbance bands were observed at 3420 and 1620 cm^−1^, which corresponded to the hydrogen-bonded hydroxyl (OH) and amide I. The bands found at 1620 cm^−1^ could be due to the characteristic asymmetrical stretch of the carboxylate and carbonyl groups and the characteristic peaks of aromatics C–C stretching. The peak at 1040 cm^−1^ was due to the C–O stretching vibration of the alcoholic groups. The bands at 1050 cm^−1^ indicated the presence of C–O stretching of alcohols, carboxylic acids, ester and ether groups. The size of the particle derived from the biological method shows an average size of 5 nm (Figure 1D). Transmission electron microscopy (TEM) images of the PdNPs showed that all of the particles were spherical in shape, dispersed within a range of 5–20 nm, and had an average particle size of 5 nm (Figure 1E,F); the images matched the dynamic light scattering (DLS) data exactly. All of the characterization data were significantly consistent with earlier reports using tea extract and leaf extract of *Anacardium occidentale*, *Pulicaria glutinosa* and *Evolvulus alsinoides* [30,42,43,44].

### 2.2. Trichostatin A (TSA) and PdNPs Inhibit Breast Cancer and HeLa Cell Viability

**Figure 2:**
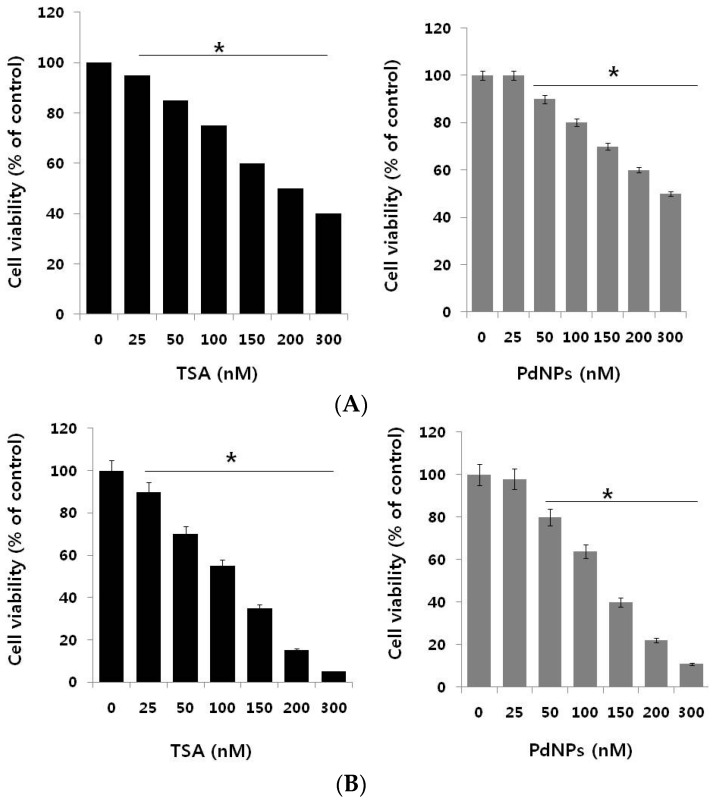
The dose-dependent effect of trichostatin A (TSA) and PdNPs on cell viability in human breast and cervical cancer cells. (**A**) The human breast cancer cells (MDA-MB-231) were incubated with various concentrations of TSA (0–300 nM) or PdNPs (0–300 nM), for 24 h. Cell viability was measured by WST-8; (**B**) Human cervical cancer cells were incubated with various concentrations of TSA (0–300 nM) or PdNPs (0–300 nM) for 24 h. Cell viability was measured using WST-8. The results are expressed as the mean ± standard deviation of three separate experiments. The treated groups showed statistically-significant differences from the control group, as determined by Student’s *t*-test (* *p* < 0.05).

The potential cytotoxic effect of TSA and PdNPs in breast and cervical cancer cells was evaluated. First, we examined their inhibitory potential on the growth of the MCF-7 breast cancer cell line. Cells were treated with different concentrations of TSA (25–300 nM) and PdNPs (25–300 nM) for 24 h, and cell viability was measured using the WST-8 (5-(2,4-Disulfophenyl)-3-(2-methoxy-4-nitrophenyl)-2-(4-nitrophenyl)-2*H*-tetrazolium) assay. A significant inhibitory effect of TSA was measured from 25–300 nM and PdNPs at 50–300 nM. Figure 2A shows that the MCF-7 cell viability decreased significantly in a dose-dependent manner. MCF-7 cells treated with either 200 nM of TSA or 300 nM of PdNPs had a 50% reduction in cell viability (Figure 2A). The IC_50_ values for TSA and PdNPs in MCF-7 cells were 200 and 300 nM, respectively. Vigushin et al. [45] demonstrated the anti-proliferative activity and anti-tumor activity of TSA in eight breast carcinoma cell lines with an IC_50_ value between 26.4 and 308.1 nM and in carcinogen-induced rat mammary cancer model, respectively.

Next, we examined the dose-dependent effect of TSA or PdNPs on cervical cancer cells. TSA and PdNPs inhibited the survival of cervical cancer cells in a concentration-dependent manner. The cytotoxic effects of TSA were more pronounced, compared to those of PdNPs. TSA, at a 100 nM concentration, inhibited cervical cancer cell viability by approximately 50%, whereas 125 nM PdNPs inhibited the viability by approximately the same percentage (Figure 2B). TSA exhibited a stronger toxic effect than PdNPs. Wu et al. [46] reported that HeLa cells treated with lower concentrations of TSA (0.1–1.0 µM) slightly activated cell growth within 12 h. Then, it marginally suppressed cell growth, but did not induce cell death after 24 h. An increased TSA concentration (1.0 and 2.0 µM) completely inhibited cell growth after 24 h of treatment. Our results are consistent with this report. We demonstrated that TSA inhibited cervical cancer cell growth in a dose- and time-dependent manner [47]. Yan et al. [6] demonstrated that a combination of curcumin and TSA enhanced anticancer effects in breast cancer cells by decreasing cell viability. Recently, we reported that PdNPs effectively induced cell death in ovarian cancer cells by decreasing cell viability in a dose-dependent manner [30]. The combined data suggest that either TSA or PdNPs effectively and significantly decreased cervical cancer cell viability to a greater degree than breast cancer cells. Therefore, further experiments were focused on HeLa cells.

### 2.3. A Combination of TSA and PdNPs Dose-Dependently Inhibits HeLa Cell Viability

**Figure 3:**
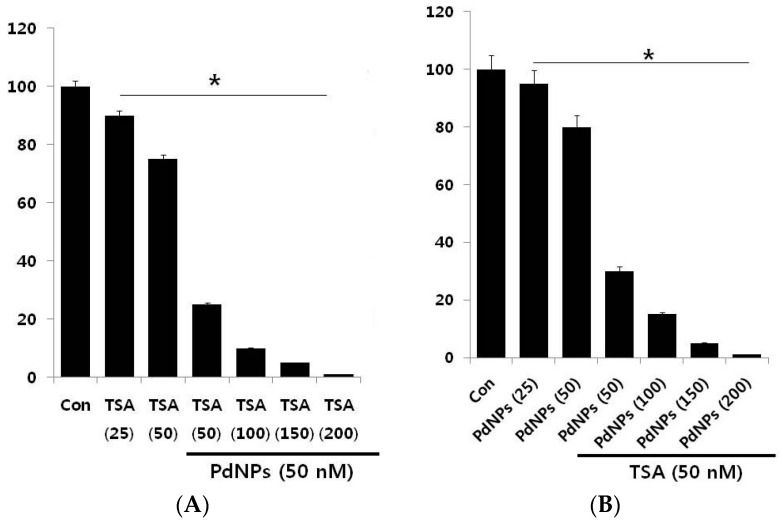
Increasing concentrations of TSA or PdNPs enhance the loss of cell viability in human cervical cancer cells. (**A**) Human cervical cancer cells were co-incubated, for 24 h, with increasing concentrations of TSA (50–200 nM) and 50 nMPdNPs or increasing concentrations of PdNPs (50–200 nM) and 50 nM TSA (**B**). The results are expressed as the mean ± standard deviation of three separate experiments. The treated groups showed statistically-significant differences from the control group, as determined by the Student’s *t*-test (* *p* < 0.05). Con: Control.

The effective combined cytotoxic dose was examined by simultaneously adding TSA (50–200 nM) and a fixed concentration of PdNPs (50 nM) to HeLa cells. The results showed that increasing concentrations of TSA with PdNPs significantly reduced cell viability, compared to singular treatment (Figure 3A). Similarly, we examined a combination of increasing concentrations of PdNPs (from 50–200 nM) and a fixed concentration of TSA (50 nM). The results suggested that the increasing concentration of PdNPs significantly influenced the combinatorial effect, which was comparable to the effect of increasing the TSA concentration. Notably, an increased concentration of TSA from 50–200 nM, in combination with 50 nM PdNPs, further inhibited the HeLa cell growth (Figure 3B). The higher concentration of TSA and PdNPs caused a higher cytotoxic effect; therefore, we selected a combination of TSA (50 nM) and PdNPs (50 nM). This was obviously a better strategy to improve the anticancer activity of PdNPs, in HeLa cells. Therefore, the remaining experiments were carried out in cells treated with a combination of TSA (50 nM) and PdNPs (50 nM), unless specified otherwise. Previous studies reported that a combination of TSA and curcumin produced significant anti-proliferative and apoptotic effects than either agent alone [6]. A combination of quercetin (5 µM) and 82.5 nM of TSA significantly increased the cytotoxic effect in A549 cells [48].

### 2.4. The Combination of TSA and PdNPs Inhibits Cell Viability and Histone Deacetylase (HDAC) Activity

**Figure 4:**
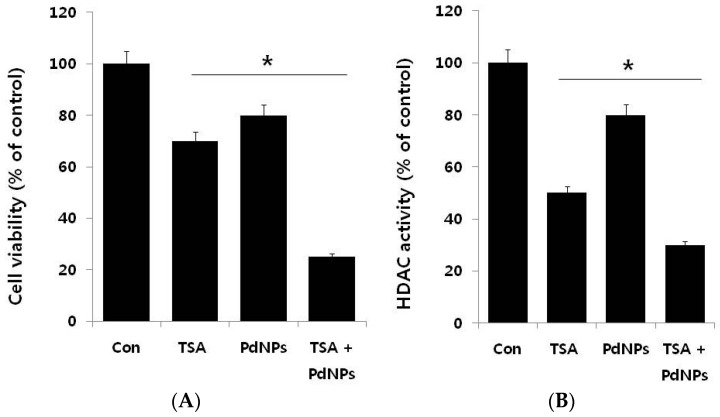
The effect of TSA or PdNPs alone or the combinatorial effect of TSA and PdNPs on cell viability and HDAC activity, in human cervical cancer cells. Human cervical cancer cells were incubated with TSA (50 nM) or PdNPs (50 nM) or both TSA (50 nM) and PdNPs (50 nM) for 24 h. (**A**) Cell viability was measured using WST-8; (**B**) HDAC activity was measured.

The next series of experiments addressed the question of whether there is a synergistic effect of TSA and PdNPs on HeLa cell cytotoxicity. Therefore, HeLa cells were incubated with TSA (50 nM) or PdNPs (50 nM) for 24 h, either alone or in combination. HeLa cells treated with TSA had a 25%–30% decrease in cell viability, compared to the untreated control; whereas, PdNPs treatment resulted in a 20%–25% decrease in cell viability, compared to the untreated control. The viability of cells co-incubated with TSA and PdNPs decreased by 75%, compared to a 20%–30% decrease in cells treated with either TSA or PdNPs alone (Figure 4A). These results indicated a synergistic effect between TSA and PdNPs, on HeLa cell cytotoxicity. Moreover, the lower concentrations of these compounds would reduce the side effects; therefore, it is a better potential therapeutic approach. It could be a novel and effective tool to kill cancer cells effectively in a time- and dose-dependent manner. Sack et al. [49] demonstrated a synergistic cytotoxicity effect of cerium oxide nanoparticles (CNP) and doxorubicin in A375 melanoma tumor cells. The treatment consisted of 300 mmol/L CNP for 48 h, 0.5 mmol/L doxorubicin for 24 h or a combination of both [49]. Our results showed that the synergistic effect of TSA and PdNPs at 24 h of treatment was much higher than that of other nanoparticles, such as CNP [49].

We examined the effects of TSA, PdNPs or a combination of both for 24 h on the HDAC activity in HeLa cells. As shown in Figure 4B, TSA, at low concentrations, significantly inhibited higher HDAC activity, compared to PdNPs at the same concentration. The potential explanation for the specific TSA enhancement of HDAC activity could be specific targeting of HDAC, while PDNPs may inhibit HDAC activity through secondary effects. These results indicated that PdNPs had an effect on HDAC activity, but it was less than TSA. However, the TSA and PdNP combination exhibited a significant effect. The TSA inhibitory effect on HDAC activity was reported in several breast cancer cell lines, with an IC_50_ of 2.4 nM. The mechanism was through pronounced histone H4 hyperacetylation [45]. Treatment of HeLa cells with different concentrations of TSA (10–50 nM) for 72 h resulted in dose-dependent inhibition of HDAC activity by increasing the forms of acetylated histone 3 and 4 [47]. Our results were in line with previous reports in cervical cancer cells.

### 2.5. Combination of TSA and PdNPs Enhances Cytotoxicity

**Figure 5:**
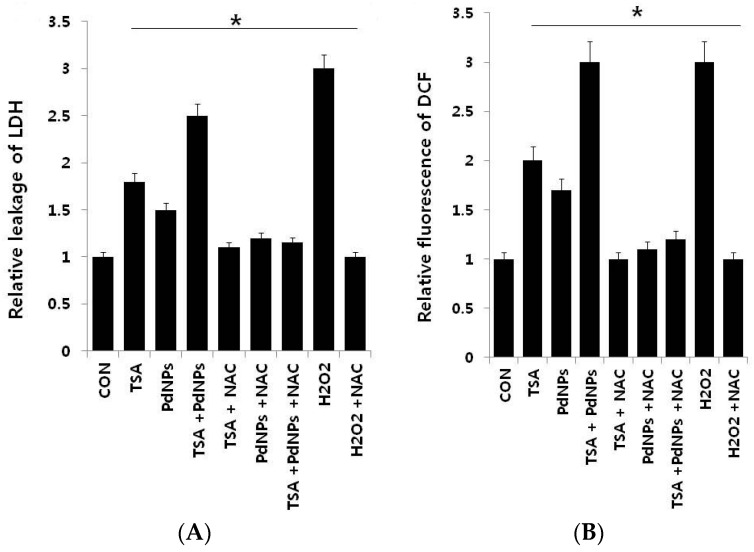
The effect, of TSA, PdNPs or a combination of TSA and PdNPs, on human cervical cancer cell cytotoxicity. The cells were treated, for 24 h, with TSA (50 nM), PdNPs (50 nM) or a combination of TSA (50 nM) and PdNPs (50 nM). (**A**) Lactate dehydrogenase (LDH) activity was measured at 490 nm, using an LDH cytotoxicity kit (Aldrich, St. Louis, MO, USA); (**B**) Reactive oxygen species were measured, as the relative fluorescence of 2′,7′-dichlorofluorescein, with a spectrofluorometer. The results are expressed as the mean ± standard deviation of three independent experiments. The treated groups showed statistically-significant differences from the control group, as determined by Student’s *t*-test (* *p* < 0.05). NAC: *N*-acetylcysteine.

Lactate dehydrogenase (LDH) is released into extracellular space when the plasma membrane is damaged. There are several cytotoxicity assays, but measurement of LDH leakage is considered to be the essential toxicity assay [30,50]. Therefore, we examined TSA and PdNPs cytotoxic effects. LDH leakage was measured in cells treated with TSA, PdNPs or a combination of TSA and PdNPs in the presence and absence of *N*-acetylcysteine (NAC) (Figure 5A), which is an antioxidant compound [51]. The data clearly indicated that all treatment groups increased LDH leakage, compared to control cells. The combination treatment increased LDH leakage to a higher level than TSA or PdNPs treatments alone. Our results are in line with previous studies, which demonstrated that submicromolar concentration of TSA, such as 1, 10 and 100 nM, increased LDH leakage in primary hepatic stellate cells [52].

Next, we examined reactive oxygen species (ROS) generation in cells treated with each of the three groups, in the presence and absence of NAC. ROS generation is a collective term for molecules generating oxidative stress, such as hydrogen peroxide (H_2_O_2_) and hydroxyl radicals (HO) [53]. The results from our data suggested that either TSA or PdNPs enhanced the generation of ROS; however, the effect was more pronounced in the combinatorial treatment. The amount of ROS was almost equal to H_2_O_2_-treated cells. Notably, NAC strongly suppressed ROS generation in TSA- or PdNP-treated HeLa cells, as well as cells treated with both TSA and PdNPs (Figure 5B). Several previous studies suggested that HDAC inhibitors induce leukemia, prostate and cervical cancer cell death through the generation of ROS [47,54,55]. PdNPs induce LDH leakage and ROS generation in ovarian cancer cells [31]. Ungerstedt et al. [56] demonstrated that oxidative stress is a critical factor in HDAC inhibitor-induced cell death [56]. Recently, several studies showed that histone deacetylase inhibitors enhance ROS production, the mitochondrial respiratory chain and are the major source of ROS production [57]. Our results showed that the combination of TSA and PdNPs has a greater effect on LDH and ROS generation, compared to singular treatments. The combinatorial treatment also induces significantly more cytotoxicity than the singular treatments.

### 2.6. Effect of TSA and PdNPs on Oxidative Stress Markers

**Figure 6:**
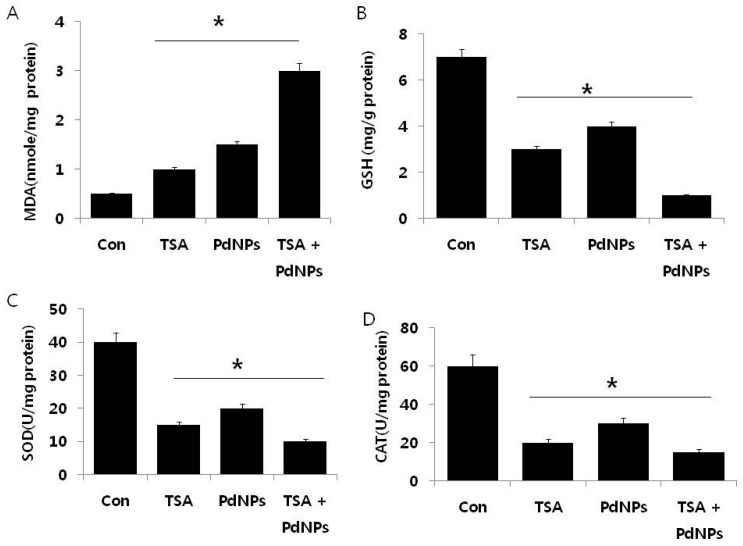
The effect of TSA, PdNPs or both TSA and PdNPs on oxidative stress markers, in human cervical cancer cells. Cells were treated for 24 h with TSA (50 nM), PdNPs (50 nM) or the combination of TSA (50 nM) and PdNPs (50 nM). (**A**) The concentration of malondialdehyde, expressed as nanomoles per milligram of protein; (**B**) the concentration of glutathione, expressed as milligram per gram of protein; (**C**) the specific activity of superoxide dismutase, expressed as units per milligram of protein; (**D**) the specific activity of catalase, expressed as units per milligram of protein. The results are expressed as the mean ± standard deviation of three independent experiments. There was a significant difference in the treated cells compared to the untreated cells, as determined by Student’s *t*-test (* *p* < 0.05).

The maintenance of ROS level could be an effective therapy for killing cancer cells, rather than normal cells, and ROS has a dual role in cell survival and death by the elevation of ROS production or a decline of ROS-scavenging capacity [57,58,59,60,61,62]. Oberley et al. demonstrated that the levels of ROS-scavenging enzymes are significantly altered in malignant cells and in primary cancer tissues [63,64]. Thus, we investigated whether TSA, PdNPs or a combination of both could influence the level of pro- and anti-oxidative markers, such as malondialdehyde (MDA), glutathione (GSH), superoxide dismutase (SOD) and catalase (CAT), in HeLa cells (Figure 6A–D).

The levels of MDA in control, TSA-treated, PdNPs-treated and TSA plus PdNPs-treated cells were 1, 1.5 and 3 nanomole/mg of protein, respectively. It was significantly higher in all three treatment groups, compared to the control. Interestingly, the combined TSA and PdNPs treatment significantly increased the MDA level (Figure 6). Previous findings suggested that ovarian cancer cells treated with PdNPs could result in an abundance of lipid peroxides, increased LDH release and increased MDA levels [30]. The level of MDA was higher in cells treated with PdNPs rather than TSA, the reason for the higher level of MDA being that PdNPs could target multiple pathways responsible for stress compared to TSA. Next, we investigated the levels of GSH, SOD and CAT in cells exposed to TSA and PdNPs (Figure 6). Their contents have become important indicators of the antioxidant capacity of cells [65]. The intracellular GSH content influences the effect of apoptosis induced by anticancer drugs [66,67]. The levels of GSH, SOD and CAT were significantly lower in TSA, PdNPs and the combinatorial treatment, compared to those in the control (Figure 6). Our results are consistent with previous studies, which demonstrated that TSA increased O_2_^•−^ and decreased GSH in HeLa cells. Huang et al. [68] described how SOD is considered to be the target for selective killing of cancer cells. Inhibition of SOD causes the accumulation of cellular O_2_^−^, which leads mitochondria-mediated apoptosis. Previous studies demonstrated that decreased catalase activity in mouse liver cancer cells is due to increasing ROS levels [69].

### 2.7. Combination of TSA and PdNPs Disrupts Membrane Potential (MMP) and Enhances Caspase-3 Activity

**Figure 7:**
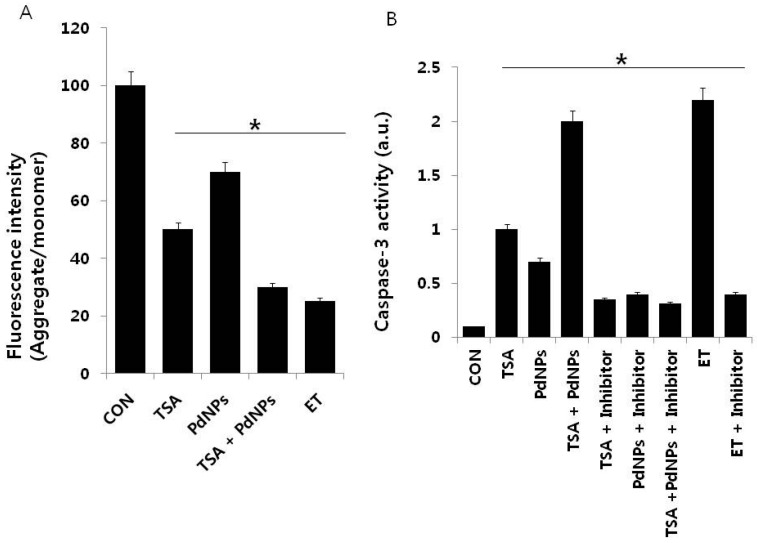
The effect of TSA or PdNPs alone or a combination of TSA and PdNPs on mitochondrial membrane potential (MMP) and caspase-3 activities. Cells were treated for 24 h with TSA (50 nM), PdNPs (50 nM) or a combination of TSA (50 nM) and PdNPs (50 nM). (**A**) MMP (ratio of JC-1 aggregate to monomer) in cervical cancer cells was determined after treatment; (**B**) the cells were treated for 24 h with TSA (50 nM), PdNPs (50 nM) or a combination of TSA (50 nM) and PdNPs (50 nM), with and without caspase inhibitor. The concentration of p-nitroanilide released from the substrate was calculated from the absorbance at 405 nm. The results are expressed as the mean ± standard deviation of three separate experiments. The treated groups showed statistically-significant differences from the control group, as determined by Student’s *t*-test (* *p* < 0.05). ET: Etoposide.

Apoptosis is mediated by an intrinsic pathway that is an essential event involved in TSA anti-tumor activity [70]. Bcl-2 family proteins play a crucial role in the disruption of mitochondrial membrane potential (MMP) and the release of cytochrome c [71]. For example, Vorinostat and TSA disrupted MMP, through increased expression of *BH3-only Bcl-2* family genes [72]. Cells were treated with TSA and PdNPs, followed by JC-1 dye measurement of MMP, to determine if the combined treatment had an effect on MMP. A dramatic decrease in the ratio of red-green fluorescence intensity was observed in cells treated for 24 h with the combination of TSA and PdNPs. The data indicated that the treatment resulted in rapid depolarization of the mitochondrial membranes with a 3–4-fold decrease in the ΔΨ_m_ (Figure 7A). These results suggested that the collapse of the ΔΨ_m_ was an early event in PdNPs-induced apoptosis [30]. The loss of MMP (ΔΨ_m_) in HeLa cells by TSA implied that TSA-induced apoptotic cell death was tightly correlated with the collapse of MMP (ΔΨ_m_) [45]. A high ratio of Bax to Bcl-2 caused the disruption of MMP (ΔΨ_m_) and apoptosis in cells [73]. Similarly, HDAC inhibitors downregulated Bcl-2 expression and induced apoptosis in many cancer cells [47,74]. Human renal carcinoma cells co-treated with TSA and TRAIL effectively induced apoptosis through loss of MMP [75]. These results supported the view that the relative loss of MMP could trigger HeLa cell apoptosis.

Caspases are cysteine proteases involved in the execution of apoptosis. The caspase-9-caspase-3 cascade is activated by pro-apoptotic molecules, such as cytochrome c released from mitochondria [76]. Therefore, we examined the involvement of caspase-3 in cells that were treated for 24 h with TSA, PdNPs or a combination of TSA and PdNPs, in the presence or absence of a caspase-3 inhibitor (Z-Asp(O-Me)-Glu(O-Me)-Val-Asp(O-Me) fluoromethyl ketone, Z-DEVD). The combination of TSA and PdNPs had a significantly higher level of caspase-3 activity, compared to cells treated with either one singularly. This indicated that the combinatorial treatment could promote cell death (Figure 7B). The elevated caspase-3 activity declined in the presence of caspase-3 inhibitor. Additionally, we used etoposide as a benchmark to show the clear involvement of caspase-3-mediated apoptosis. Similarly, caspase-3/7 activity in human vertebral-cancer of the prostate (VCaP) prostate cancer cells increased dramatically after 24-h incubation with 5 mM valproate (VPA) or 100 nM TSA [77]. The results from these experiments clearly indicated that TSA- or PdNP-induced HeLa cell apoptosis was mediated by the activation of caspase-3. Previous studies demonstrated that TSA induced apoptosis through activation of various caspase cascades, including the caspase-8 cell death receptor pathway and the caspase-9 mitochondrial pathway [47]. PdNPs induced human ovarian cancer cell apoptosis by caspase-3. Human lung cancer cells treated with TSA had increased caspase-3 activity, whereas quercetin enhanced TSA induction of caspase-3 activity by 113% [48]. Collectively, the data demonstrated that the combination of TSA with PdNPs affects HeLa cells through the activation of caspase-3.

### 2.8. Combination of TSA and PdNPs Enhances Apoptosis

**Figure 8:**
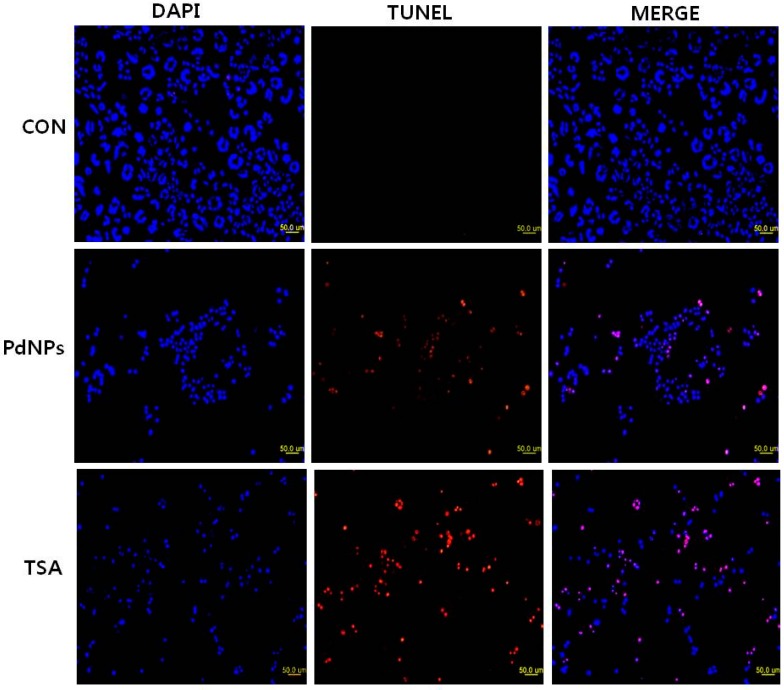

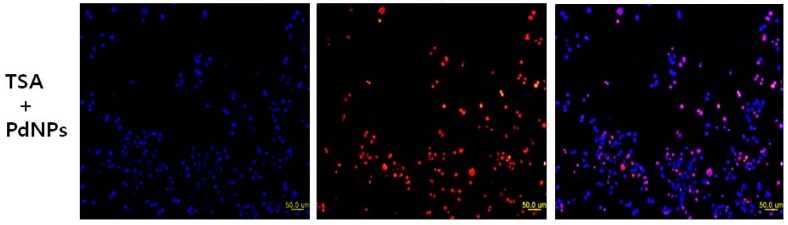
Effect of TSA or PdNPs alone or in combination on apoptosis in human cervical cancer cells. The human cervical cancer cells were treated for 24 h with TSA (50 nM), PdNPs (50 nM) or a combination of TSA (50 nM) and PdNPs (50 nM). Apoptosis was assessed in a TUNEL assay; the nuclei were counterstained with DAPI. Representative images show apoptotic (fragmented) DNA (red staining) and the corresponding cell nuclei (blue staining).

Caspase-3 activation induces DNA fragmentation, which is a biochemical hallmark of apoptosis. HeLa cells were treated with TSA, PdNPs or a combination of TSA and PdNPs for 24 h to verify their induction of apoptosis. The TUNEL assay was used to analyze their effect. The number of cells positively stained by the TUNEL reagents increased significantly with either singular or combined TSA and PdNP treatment (Figure 8). However, the combined treatment stimulated a greater level of apoptosis than the single treatments. Our results were consistent with previous studies, which demonstrated that TSA and sodium butyrate induced apoptosis by DNA fragmentation in a concentration-dependent manner by increased chromatin relaxation and enhanced accessibility of DNA in thymocytes [78]. Yee et al. [79] showed that the loss of p815 mastocytoma cell viability was due to apoptotic events, such as DNA fragmentation. TSA was not only involved in DNA fragmentation, but it also had a significant impact on arresting HeLa cells in the S phase; it eventually induced apoptosis [80]. Several studies claimed that several HDAC inhibitors and TSA induced apoptosis in HeLa cells [81,82] and eosinophils and neutrophils in the presence and absence of growth factors [83]. Piacentini et al. [84] reported that TSA, in combination with five different chemotherapeuticdrugs, induced apoptosis in 10 pancreatic adenocarcinoma cell lines; the data indicated that TSA is a suitable combinatorial agent.

### 2.9. Combination of TSA and PdNPs Upregulates Apoptotic Genes

**Figure 9:**
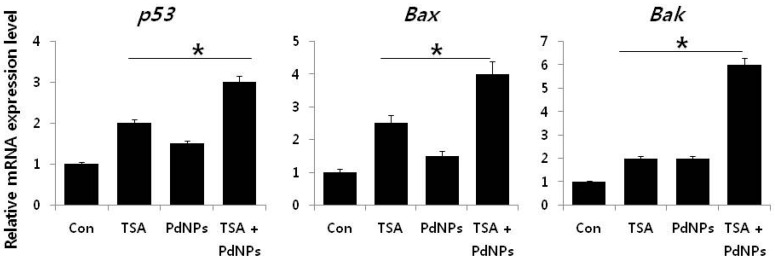

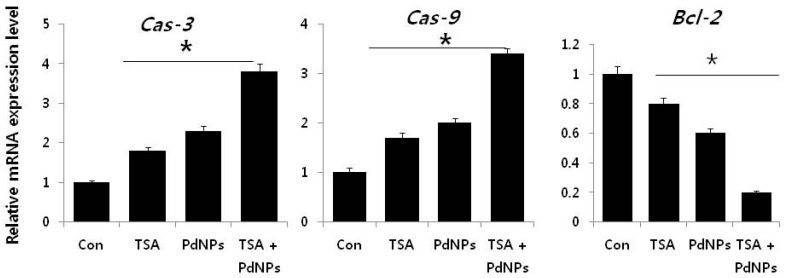
The impact of TSA, PdNPs or a combination of TSA and PdNPs on the expression of apoptotic and anti-apoptotic genes. The relative mRNA expression of apoptotic and anti-apoptotic genes was analyzed by qRT-PCR, in human cervical cancer cells treated for 24 h with TSA (50 nM), PdNPs (50 nM) or a combination of TSA (50 nM) and PdNPs (50 nM). The results are expressed as the mean ± standard deviation of three separate experiments. The treatment groups showed statistically-significant differences from the control group, as determined by Student’s *t*-test (* *p* < 0.05).

It is well known that various pro- and anti-apoptotic proteins regulate cell death pathways. Therefore, we examined the link between apoptosis and the three treatment groups, by measuring *p53*, *Bax*, *Bak*, *caspase-3/9* and *Bcl-2* mRNA levels. Cells treated with TSA, PdNPs or a combination of both had increased expression of *p53*, *Bax*, *Bak* and *caspase-3/9*, while the expression of *Bcl-2* decreased (Figure 9). However, the enhancement of TSA and PdNPs, on apoptosis-induced gene expression, was greater than that on TSA or PdNPs alone. This indicated that the combinatorial effect worked better than the single treatment [85]. Dysregulation of HDAC activity by TSA leads to silencing tumor suppressor genes, such as *p53*, which plays an important role in apoptosis [48,86,87]. Mouse tumors treated with TSA in combination with quercetin had higher *p53* and apoptosis levels, compared to the control and TSA-treated mice [48]. Wu et al. [88] recently reported that genistein, in combination with TSA, markedly increased *p53* and caspase expression in A549 cells. The data indicated that TSA plays an important role in enhancing TSA-induced apoptosis in HeLa cells via *p53*. Several HDAC inhibitors, including TSA, decreased expression Bcl-2, Bcl-xL and XIAP expression. They also enhanced the expression of pro-apoptotic proteins, such as Bax and Bak [89,90]. MCF-7 and MDA-MB-231 breast cancer cells treated with a combination of suberoylbis-hydroxamic acid (SBHA) and a proteasome inhibitor had significantly higher *p53*, Bax, Bcl-xS and Bak protein levels and decreased the Bcl-2 level [91]. A combination of TSA and TRAIL effectively increased caspase-3, -8, and -9 activation and degradation, in human RCC Caki cells [92]. Our results clearly suggested that TSA, PdNPs or the combination of TSA and PdNPs increased the expression of caspase-3 and -9. However, the combinatorial effect was greater than the single treatment. Altogether, the data from this study clearly suggested that the molecular mechanism of apoptosis induced by TSA, PdNPs or their combination may modulate the Bcl-2 family of proteins.

## 3. Materials and Methods

### 3.1. Materials

Penicillin-streptomycin, trypsin-EDTA, RPMI 1640 medium and 1% antibiotic-antimycotic were obtained from Life Technologies/Gibco (Grand Island, NY, USA). PdCl_2_, for the preparation of the PdNPs, was purchased from Sigma-Aldrich (St. Louis, MO, USA). Trichostatin A, NAC, H_2_O_2_, fetal bovine serum (FBS) and the in vitro toxicology assay kit were purchased from Sigma-Aldrich (St. Louis, MO, USA). All other chemicals were purchased from Sigma-Aldrich, unless otherwise stated.

### 3.2. Synthesis of PdNPs

The PdNPs were prepared with saponin, according to a previously-described method with some modifications [30]. Saponin (1 mg) was suspended in 90 mL of sterile distilled water, mixed well for 5 min and then used in PdNPs’ synthesis. The saponin solution was combined with 10–100 mL of a 1 mM aqueous PdCl_2_ solution, then incubated for 6 h at 60 °C, with constant stirring. The reduction reaction occurred rapidly and was indicated by a solution color change, from light to bright brown.

### 3.3. Characterization of PdNPs

The synthesized PdNPswere characterized according to the method described previously [30].

### 3.4. Cell Culture

Human MCF-7 breast cancer cells and cervical adenocarcinoma HeLa cells were from Dr. Zhang’s laboratory stock. Both human cancer cells were maintained in a humidified incubator at 5% CO_2_ and 37 °C. HeLa cells were cultured in RPMI-1640 (Sigma-Aldrich, St. Louis, MO, USA), supplemented with 10% fetal bovine serum (FBS; Sigma-Aldrich) and 1% penicillin-streptomycin (Gibco BRL, Grand Island, NY, USA). Cells were routinely grown in 100-mm plastic tissue culture dishes (Nunc, Roskilde, Denmark) and harvested with a solution of trypsin-EDTA, while in a logarithmic phase of growth.

### 3.5. Cell Viability Assay

The WST-8 assay was performed, as previously described [30]. Typically, 2 × 10^5^ cells were seeded into a 96-well plate and cultured, at 37 °C under 5% CO_2_ for 24 h, in RPMI-1640 standard medium, supplemented with 10% FBS. Next, the cells were washed twice with 100 µL of serum-free RPMI-1640 and incubated, for 24 h, with 100 µL of media, containing TSA (25–300 nM) or PdNPs (25–300 nM). Cells that were not exposed to TSA or PdNPs served as controls. The cells were washed twice with serum-free RPMI-1640, after 24 h of exposure. WST-8 solution (15 µL) was added to each well, which contained 100 µL of serum-free RPMI-1640. The mixture (80 µL) was transferred to another 96-well plate, after 1-h incubation at 37 °C under 5% CO_2_. Absorbance was measured at 450 nm, using a microplate reader.

### 3.6. Histone Deacetylase Activity

Histone deacetylase activity was assayed, as described [93]. HeLa cells were treated with TSA, PdNPs or in combination for 24 h. The cells were washed in PBS and suspended in 5 volumes of lysis buffer (R&D Systems, Inc., Minneapolis, MN, USA). Next, cells were harvested, and whole cell protein was extracted, using RIPA lysis buffer. Protein concentrations were measured, using BCA kit reagents. Supernatant samples, containing 20 µg of total protein, were used to assay HDAC activity. The samples and HDAC substrate, provided by the assay kit, were added to each well of a 96-well microtiter plate and incubated at 37 °C for 1 h. HDAC activity was measured using the HDAC Activity Assay kit (Sigma-Alrich, St. Louis, MO, USA). Experimental procedures were performed, according to the manufacturer’s instructions.

### 3.7. Cytotoxicity Assay

The integrity of the human ovarian cancer cell membrane was evaluated by measuring the cellular release of lactate dehydrogenase (LDH), according to the manufacturer’s instructions (in vitro toxicology assay kit, TOX7, Sigma-Alrich, St. Louis, MO, USA) and as described previously [18]. Briefly, the cells were exposed to each of the 3 treatment groups, for 24 h, and then LDH was measured. Additionally, cells from each treatment group were incubated, with or without 2 mM NAC.

Reactive oxygen species (ROS) were estimated, according to a method described previously [18]. The cells were seeded into 24-well plates, at a density of 5 × 10^4^ cells per well and cultured for 24 h. They were washed twice with phosphate-buffered saline (PBS) before adding fresh media, containing each of the 3 treatment groups, then incubated for 24 h. Cells were also incubated with the same treatment groups with or without 2 mM NAC. The cells were then supplemented with 20 µM DCFH-DA; the incubation continued for 30 min at 37 °C. The cells were rinsed with PBS before adding 2 mL of PBS to each well. Fluorescence intensity was determined, using a spectrofluorometer (Gemini EM, CA, USA), with excitation at 485 nm and emission at 530 nm.

### 3.8. Measurement of Oxidative Stress Markers

Oxidative stress markers, such as malondialdehyde (MDA), glutathione (GSH), superoxide dismutase (SOD) and catalase (CAT), were assayed with reagents from various kits, according to each manufacturer’s instructions (Sigma). Briefly, the cells were cultured in 75 cm^2^ culture flasks and exposed to TSA, PdNPs or a combination of both, for 24 h. The cells were harvested in chilled PBS, by scraping and washing twice with 1× PBS at 4 °C for 6 min at 1500 rpm. The cell pellet was sonicated at 15 W for 10 s (3 cycles) to obtain the cell lysate. The resulting supernatant was stored at 70 °C, until analyzed.

### 3.9. Measurement of Mitochondrial Membrane Potential

Briefly, the cells were cultured in 75-cm^2^ culture flasks and exposed to TSA, PdNPs or a combination of both, for 24 h. MMP was measured, as described previously, using a cationic fluorescent indicator JC-1 (Molecular Probes, Eugene, OR, USA) [30]. JC-1 is a lipophilic cation, which, in a reaction driven by ΔΨ_m_ in normal polarized mitochondria, assembles into a red fluorescence-emitting dimer, forming JC-1-aggregates. Cells were incubated with 10 µM JC-1 at 37 °C for 15 min, washed with PBS and resuspended in PBS; then, fluorescence intensity was measured. MMP was expressed as the ratio of the fluorescence intensity of the JC-1 aggregates to monomers.

### 3.10. Measurement of Caspase-3 Activity and TUNEL Assay

The caspase-3 and TUNEL assays were performed, according to the methods described earlier [18]. The cells were treated with each of the 3 experimental groups, in the presence of a caspase-3 inhibitor, for 24 h. Caspase-3 activity was measured in the cancer cells, according to the manufacturer’s instructions in a kit from Sigma-Aldrich (St. Louis, MO, USA).

Apoptosis was examined in cells treated with all 3 groups, for 24 h, using a DNA Fragmentation Imaging Kit (Roche, Mannheim, Germany), following the manufacturer’s instruction. The culture medium was aspirated after the incubation period, and the cell layers were trypsinized. The detached cells were reattached on 0.01% polylysine-coated slides, fixed with 4% methanol-free formaldehyde solution, and stained, according to the manufacturer’s instructions for the TUNEL protocol.

### 3.11. Extraction and Amplification of mRNA

Total RNA was extracted from cells, treated with TSA, PdNPs or a combination of both for 24 h, using an Arcturus picopure RNA isolation kit (eBioscience, San Diego, CA, USA); samples were prepared according to the manufacturer’s instructions. Real-time RT-PCR was conducted using a Vill7 (Applied Biosystems, Foster City, CA, USA) and SYBR Green, as the double-stranded DNA-specific fluorescent dye (Applied Biosystems) Target gene expression levels were normalized to *GAPDH* expression, which was unaffected by treatment. The RT-PCR primer sets are shown in Table 1. RT-PCR was performed independently in triplicate, for each of the different samples; the data are presented as the mean values of gene expression measured in treated samples versus the control.

### 3.12. Statistical Analyses

All assays were conducted in triplicate, and each experiment was repeated at least 3 times. The results represent the mean of at least 3 independent experiments (mean ± SD). Student’s *t*-test or one-way analysis of variance (ANOVA), followed by Tukey’s test for multiple comparisons, were calculated, using the Graph-Pad Prism software (GraphPad Software, San Diego, CA, USA). The differences were considered significant at *p* < 0.05.

## 4. Conclusions

Cervical cancer is the leading cause of cancer death in women worldwide, which accounts for 7.5% of all female cancer-related deaths; however, it is predominant in certain ethnic and socio-economic groups. Therefore, we hypothesized that TSA, together with PdNPs, may be an effective nanoparticle-mediated chemotherapy, which could significantly inhibit cancer cell viability. We assessed the effects, of all three treatments, in human cervical cancer cells via a series of biochemical assays. This is the first study to show the combinatorial effect of TSA and PdNPs in cervical cancer cells. The fact that the combination treatment resulted in a significant reduction of cell viability, increased oxidative stress, loss of MMP and enhanced caspase-3/9 activity suggests that cervical cancer cells become more sensitive to lower doses of PdNPs, when treated with TSA. It is important to focus on the synergistic cytotoxicity effects and the oxidative stress measured in the combination treatment. Furthermore, our results show that the apoptotic responses, induced by the three treatments, are a result of upregulated pro-apoptotic and downregulated anti-apoptotic proteins. The potential mechanisms, stimulated by the combinatorial treatment, are the modulation of p53 and Bcl-2 family proteins. This finding suggests that cells treated with TSA plus PdNPs experienced significantly higher toxicity, compared to cells treated with TSA or PdNPs alone. Our data supports a strong synergistic interaction between TSA and PdNPs in the human cervical cancer cell line that we used. This approach could be an alternative approach for women experiencing chemoresistance; it may, also, free them from any side effects. Our data suggest that the combination of TSA and PdNPs could provide a novel, effective, supplemental treatment for cervical cancer patients.

## Figures and Tables

**Table 1:** Primers used for quantitative real-time polymerase chain reaction for analysis of apoptotic, and anti-apoptotic, gene expression.

Serial Number	Gene	Direction	Primers(5′–3′)
1	*Bax*	F	GAG AGG TCT TTT TCC GAG TGG
		R	GGA GGA AGT CCA ATG TCC AG
2	*P53*	F	AGG AAA TTT GCG TGT GGA GTA T
		R	TCC GTC CCA GTA GAT TAC CAC T
3	*Bak*	F	CTC AGA GTT CCA GAC CAT GTT G
		R	CAT GCT GGT AGA CGT GTA GGG
4	*CAS3*	F	CAT ACT CCA CAG CAC CTG GTT A
		R	ACT CAA ATT CTG TTG CCA CCT T
5	*CAS9*	F	ACT TTC CCA GGT TTT GTT TCC T
		R	GAA ATT AAA GCA ACC AGG CAT C
6	*Bcl2*	F	CTG AGT ACC TGA ACC GGC A
		R	GAG AAA TCA AAC AGA GGC CG
